# Does High-Speed Rail Opening Affect the Health Care Environment?–Evidence From China

**DOI:** 10.3389/fpubh.2021.708527

**Published:** 2021-06-09

**Authors:** Cai-Xia Song, Cui-Xia Qiao, Jing Luo

**Affiliations:** School of Economics, Shandong Normal University, Jinan, China

**Keywords:** high-speed rail opening, health care environment, China, difference-in-differences method, economic development

## Abstract

Using the panel data of 280 prefecture-level cities in China from 2004 to 2014, this paper examines the effects of high-speed rail opening on health care environment based on Difference-in-Differences method (DID). Through an empirical analysis, the results proved that high-speed rail opening can significantly promote the health care environment and this effect is different in regions with different levels of economic development. Finally, we tested the mechanisms of how the high-speed rail opening affects the healthcare environment. High-speed rail opening improves the healthcare environment by increasing road accessibility and promoting economic development. Our results support the view that high-speed rail opening has an important contribution to the improvement of health care conditions.

## Introduction

In response to the huge demand for cross-regional movement of people and materials brought about by economic development, China has placed a high priority on transport infrastructure development. High-speed railway, with its high speed and wide coverage, can meet the need for efficient and convenient transport. As a transport infrastructure with large-scale investment, high-speed railways have a close relationship with economic development and national living standards, and also play an important role in improving the health care conditions of cities along the route (1–3).

Road infrastructure is considered to be a key catalyst for regional economic growth and residential environment (4–7). Existing studies have categorized the impacts of road infrastructure into two types: direct and indirect. Direct impacts are about service and accessibility improvements, including market opening, foreign investment and urban expansion. Indirect impacts are related to regional responses and policy changes along road construction, including greater urban attractiveness, lower development costs and tax incentives. It's also the way in which high speed rail affects the health care environment (8). Transport infrastructure can improve the healthcare environment by increasing the accessibility of healthcare resources and the use of healthcare services, thereby, improving the health of the population (9–12).

Studies have proven that the opening of a high-speed railway will have an overall impact on the development of a region (13). In the specific area of healthcare, the question of how the high-speed railway will affect the medical environment and how the advantages of the high-speed railway can be transformed into development momentum to drive healthcare development needs to be carefully considered. To address these issues, this paper uses the panel data of 280 prefecture-level cities in China from 2004 to 2014, and examines the effects of high-speed rail opening on health care environment based on Difference–in– Differences method (DID). The results of the paper show that the opening of high-speed rail is conducive to improving the health care environment. As an important part of modern transport infrastructure, high-speed rail can provide healthcare resources and health protection to regions by reducing time costs, increasing the mobility of healthcare resources and promoting economic development. The health effects of the high-speed rail opening have already been demonstrated. In addition, the effect of the opening of the high-speed rail in improving the healthcare environment is different in different regions.

The rest of the paper is organized as follows. Section Research Hypothesis proposes theoretical mechanisms and research hypotheses. Section Methodology and Data elaborates model setting and variable selection. Section Empirical Results and Analysis provides regression results and explanations. Section Robust Test provides parallel trend and endogeneity test. Section Conclusions concludes.

## Research Hypothesis

According to the new economic geography theory, which introduces spatial distance and transport costs into the economic model analysis, Krugman (14) points out that the reduction in transport costs can generate agglomeration effects of factors and industries, thus creating economies of scale and promoting economic growth.

As an important transport infrastructure in modern society, high-speed rail has the function of shortening spatial distances and reducing transport costs (15, 16). On the one hand, the agglomeration of medical industries makes it more convenient for people to access medical and health services. And on the other hand, the opening of high-speed rail makes advanced medical technology and medical equipment more easily accessible, thus improving the medical environment in backward areas (17–20).

Furthermore, the opening of the high-speed railway will facilitate the flow of talents, making it easier for specialists to communicate, expanding the geographical mobility of medical personnel and strengthening inter-regional medical cooperation (21, 22). At the same time, in order to enhance the location and competitiveness of the opening areas, the local governments of the opening areas will also improve their healthcare environment and increase their financial expenditure on healthcare (23, 24), thus improving the healthcare conditions in the opening areas and further promoting the mobility of healthcare professionals (25). Therefore, the following hypothesis is proposed.

H1: The opening of the high-speed rail will improve regional health care.

In addition, the impact of the high-speed rail on the different regions varies, as there are significant differences in economic development and infrastructure in different regions (26–28). Due to the higher level of economic development in the eastern region and its natural geographical advantage, it is beneficial for the introduction of technology and the exchange of talents. In contrast, the density of high-speed rail in the western region is currently much lower than in the eastern region due to poor infrastructure. The effect of high speed rail on improving medical conditions in the western region may be relatively limited (29–31). Therefore, the following hypothesis is proposed.

H2: The impact of high-speed rail on the healthcare environment is different in different regions.

## Methodology and Data

### Model Setting and Variable Selection

High-speed rail is opened by region and year by year. This paper uses DID model to examine the impact of high-speed rail opening on the health care environment. Regions with high-speed rail service were used as the experimental group (takes a value of 1), while others were used as the control group (takes a value of 0). By comparing the change in medical conditions between the control group and the experimental group, it is possible to identify whether the high-speed rail has improved the health care environment. The econometric model was set up as follows.

(1) $$\begin{array}{r} HCE_{it}=\alpha +\beta_{0}HSR_{it}*TIME_{it}+\beta_{1}Control_{it}+\varphi_{i}+\gamma_{t}+\varepsilon_{it}\; \end{array}$$

In the above model (1), *i* denotes prefecture-level cities, and *t* denotes years. φ_*i*_ is individual fixed effects, γ_*t*_ is time fixed effects and ε_*it*_ is random error term. *HSR* represents the high-speed rail opening. *TIME* is a time dummy variable, if a city opens a high speed rail in year *t*, then *TIME* takes the value 1 for the city in year *t* and later, and 0 for the year before. *HCE* represents health care environment. The health care environment consists of three main components: the level of medical technology (*TEC*), medical infrastructure (*MI*) and the medical workforce (*MW*). As the introduction of high-tech medical equipment is limited by data collection, the share of government expenditure on technology in government expenditure is used to represent the general level of regional medical technology. And medical infrastructure is represented by the number of regional hospital beds, medical workforce is represented by the number of regional doctors.

*Control* is a set of control variables, including (a). regional economic development (*lnGDP*), measured as the logarithm of GDP per capita; (b). openness (*FDI*), measured as the proportion of total foreign investment utilized to GDP; (c). government size (*Gov*), measured as the proportion of government expenditure to GDP; (d). human capital (*CAP*), measured as the proportion of university students to the total population; (e). regional transport infrastructure development (*lnRoad*), measured as the logarithm of regional transport miles; (f). financial development (*FIN*), measured as the proportion of deposits in financial institutions to GDP.

### Data Source

Using the panel data of 280 prefecture-level cities in China from 2004 to 2014, this paper examines the effects of high-speed rail opening on health care environment. Data on the high-speed rail opening is from the website of the National Railway Administration. Other research data is from the China Regional Economic Statistical Yearbook (2004–2014) and the China Urban Statistical Yearbook (2004–2014).

## Empirical Results and Analysis

### Benchmark Test

Table 1 shows the benchmark regression results. Columns (1) to (3) do not control for fixed effects, and columns (4) to (6) control for time and individual fixed effects. The results show that the estimated coefficient of HSR^*^TIME is significantly positive in both regressions. It indicates that the local health care environment in cities with high-speed rail opening is significantly enhanced relative to cities without high-speed rail opening, which confirms the research hypothesis 1: The opening of the high-speed rail will improve regional health care.

**Table 1:** Benchmark test.

**Variable**	**(1)**	**(2)**	**(3)**	**(4)**	**(5)**	**(6)**
	**TEC**	**MI**	**MW**	**TEC**	**MI**	**MW**
HSR*TIME	0.281^***^	0.811^***^	0.426^***^	0.164^***^	0.764^***^	0.311^***^
	(0.094)	(0.030)	(0.061)	(0.007)	(0.063)	(0.041)
LnGDP	1.470^***^	2.322^***^	3.211^*^	2.101^***^	1.860^***^	3.006^*^
	(0.143)	(0.064)	(1.829)	(0.099)	(0.217)	(1.513)
FDI	0.786^***^	10.551^***^	2.415	0.821^***^	12.639^***^	3.106
	(0.143)	(2.886)	(2.988)	(0.201)	(3.006)	(3.118)
GOV	0.101^***^	0.207^***^	0.115^*^	0.115^***^	0.194^***^	0.104^*^
	(0.001)	(0.011)	(0.026)	(0.008)	(0.019)	(0.029)
CAP	2.032^***^	0.032	1.809^***^	1.883^***^	0.009	1.775^***^
	(0.016)	(0.147)	(0.002)	(0.044)	(0.088)	(0.004)
LnRoad	1.098^**^	1.420^***^	2.042^***^	1.102^**^	1.311^***^	2.008^***^
	(0.166)	(0.094)	(0.102)	(0.179)	(0.056)	(0.123)
FIN	0.852^*^	0.971^*^	0.639^*^	0.911^*^	0.842^*^	0.597^*^
	(0.411)	(0.316)	(0.216)	(0.322)	(0.216)	(0.189)
Constant	6.874^***^	10.477	11.612	7.012^***^	8.991	3.557
	(0.622)	(13.009)	(10.998)	(2.004)	(9.106)	(6.246)
Fixed effects	NO	NO	NO	YES	YES	YES
Obs	2,890	2,890	2,890	2,890	2,890	2,890
*R*^2^	0.770	0.811	0.901	0.792	0.672	0.955

*Cluster robust standard error in parenthesis. Significance*:

* *p < 0.1*,

** *p < 0.05*,

*** *p < 0.01*.

Regarding the coefficient of control variables, it can be stated that: economic development, market openness, government size, human capital, transport infrastructure development and financial development all have a positive effect on the local health care environment at least at the 10% significance level.

### Regional Test

The differences in economic characteristics, cultural customs and resource endowments of different regions may lead to differences in the impact of the high-speed rail opening on the regional healthcare environment. Therefore, the sample was divided into three parts, East, Central and West, according to the regional classification criteria used in most of the literature. A sub-sample regression based on Equation (1) was then conducted to analyze whether there was regional heterogeneity in the impact of the high-speed rail opening on the health care environment, and the results are shown in Table 2.

**Table 2:** Regional test.

**Variable**	**(1) Eastern region**			**(2) Central region**			**(3) Western region**		
	**TEC**	**MI**	**MW**	**TEC**	**MI**	**MW**	**TEC**	**MI**	**MW**
HSR*TIME	0.811^***^	1.014^***^	1.622^***^	0.404^***^	0.923^***^	0.845^**^	1.004	0.894	0.659
	(0.009)	(0.011)	(0.019)	(0.012)	(0.043)	(0.117)	(2.334)	(0.965)	(0.880)
LnGDP	2.110^***^	3.047^***^	2.697^***^	1.601^***^	2.908^***^	2.028^*^	1.720^*^	2.910	2.661
	(0.142)	(0.176)	(0.096)	(0.116)	(2.001)	(1.004)	(0.670)	(3.311)	(4.021)
FDI	0.930^***^	1.760^***^	2.401^***^	1.212^**^	3.462^*^	2.721^*^	3.001	2.954	1.365
	(0.021)	(0.124)	(0.101)	(0.169)	(1.351)	(1.019)	(3.097)	(3.001)	(2.019)
GOV	1.116^***^	0.943^***^	0.771^*^	1.730^***^	0.891^***^	0.541^*^	0.943^***^	1.402^*^	0.673^*^
	(0.009)	(0.005)	(0.332)	(0.011)	(0.010)	(0.219)	(0.119)	(0.614)	(0.299)
CAP	1.009^***^	2.112	0.923^***^	1.304^***^	1.559	0.752^**^	2.003	0.996	1.007
	(0.106)	(3.288)	(0.007)	(0.178)	(2.110)	(0.252)	(3.019)	(1.035)	(1.984)
LnRoad	0.762^***^	0.991^***^	0.753^***^	0.711^***^	0.810	0.973	1.001	1.029	0.665
	(0.080)	(0.058)	(0.081)	(0.062)	(0.902)	(1.102)	(1.978)	(2.009)	(0.806)
FIN	1.079^***^	0.780^***^	0.881^***^	1.003^***^	0.769^***^	0.562^***^	0.922	0.832	1.014
	(0.016)	(0.008)	(0.017)	(0.012)	(0.071)	(0.018)	(1.010)	(0.901)	(1.562)
Constant	11.103^***^	9.211^***^	8.877	9.141^***^	7.943^***^	10.210	6.976^**^	7.665	9.449
	(0.266)	(2.016)	(9.041)	(1.551)	(1.032)	(11.340)	(2.114)	(8.402)	(10.152)
Fixed effects	YES	YES	YES	YES	YES	YES	YES	YES	YES
Obs	1,047	1,047	1,047	954	954	954	889	889	889
R^2^	0.411	0.512	0.572	0.694	0.560	0.811	0.667	0.681	0.591

*Cluster robust standard error in parenthesis. Significance*:

* *p < 0.1*,

** *p < 0.05*,

*** *p < 0.01*.

The regression coefficient for HSR^*^TIME is significantly positive in both the East and Central regions, but not in the West, and the coefficient for the Eastern region is greater than that for the Central region. The results suggest that the improvement of the health care environment in the central and western regions is influenced by other complex factors, as well as possibly by the density of high speed railways. What is certain is that as the level of regional economic development increases, the contribution of high-speed rail to the health environment is strengthening. Thus, the opening of high-speed railways has a greater promotional effect on the health environment in more economically developed regions. This verifies the research hypothesis 2: The impact of high-speed rail on the healthcare environment is different in different regions.

### Mechanism Test

As mentioned in Section Research Hypothesis above, the impact of the opening of the high-speed rail on the healthcare environment has two main mechanisms: (a). increasing road accessibility (free movement of healthcare resources and talent), and (b). promoting regional economic development and thus improving healthcare standards. Firstly, the article tests the first mechanism using the interaction term between regional product flows (imports and exports) and the opening of the high-speed rail. And then, the second mechanism is tested using the interaction term between regional GDP per capita and the opening of the high-speed rail. On the basis of model (1), the model for the mechanism test is set up as follows.

(2) $$\begin{array}{r} HCE_{it}=\alpha +\beta_{0}Mech_{it}{+\beta }_{1}HSR_{it}*Mech_{it}+\beta_{2}Control_{it}+\varphi_{i}\\ \;+\gamma_{t}+\varepsilon_{it} \end{array}$$

In the above Equation (2), *Mech* is the two mechanism factors to be tested (product flows and economic development). And the regression results are shown in Table 3. From columns (1) to (3), the regression coefficients for HSR^*^Product are positive, indicating that the opening of the high-speed rail enhances the health care environment by improving road accessibility and facilitating factor mobility. From column (4) to column (6), it can be concluded that the regression coefficient of HSR^*^GDP Percapita is significantly positive, indicating that the opening of high-speed rail does enhance the health care environment by promoting economic development. It can be seen that economic development is the guarantee for the health effect of the opening of the high-speed rail. In summary, both mechanisms are valid.

**Table 3:** Mechanism test.

**Variable**	**Road accessibility**			**Economic development**		
	**(1)**	**(2)**	**(3)**	**(4)**	**(5)**	**(6)**
	**TEC**	**MI**	**MW**	**TEC**	**MI**	**MW**
Product	0.049^*^	0.167^**^	0.188^**^			
	(0.020)	(0.034)	(0.062)			
HSR*Product	1.566^***^	2.100^***^	1.304^***^			
	(0.107)	(0.049)	(0.056)			
GDP per capita				0.661^*^	0.480^***^	0.532^***^
				(0.243)	(0.117)	(0.029)
HSR*GDP Percapita				1.212^***^	1.703^***^	1.005^***^
				(0.009)	(0.043)	(0.089)
Control variables	YES	YES	YES	YES	YES	YES
Fixed effects	YES	YES	YES	YES	YES	YES
Obs	2,890	2,890	2,890	2,890	2,890	2,890
*R*^2^	0.344	0.276	0.414	0.378	0.430	0.259

*Cluster robust standard error in parenthesis. Significance*:

* *p < 0.1*,

** *p < 0.05*,

*** *p < 0.01*.

## Robust Test

### Parallel Trend Test

The parallel trend assumption is an important premise of the DID model, which requires a common trend in the level of healthcare in the experimental and control groups before the opening of the high-speed rail. A “counterfactual test” of the effect of the opening of the high-speed rail was carried out by advancing the opening of the high-speed rail by 1, 2, and 3 years, respectively. If there is no significant effect of the high-speed rail on the healthcare environment, then the control and experimental samples meet the parallel trend and the results are robust. Otherwise, the findings are not credible. The results (Table 4) reported that interactive variable (HSR^*^TIME) is non-significant, verifying that the effect of the high-speed rail opening on healthcare environment in the original model is robust.

**Table 4:** Parallel trend test.

**Variable**	**(1)**	**(2)**	**(3)**
	**TEC**	**MI**	**MW**
HSR*TIME_before1	0.064	0.192	−0.154
	(0.120)	(0.330)	(−0.249)
HSR*TIME_before2	0.881	1.972	1.001
	(2.001)	(3.664)	(2.341)
HSR*TIME_before3	0.962	−4.012	1.760
	(2.176)	(−4.943)	(3.087)
Control variables	YES	YES	YES
Fixed effects	YES	YES	YES
Obs	2,890	2,890	2,890
*R*^2^	0.433	0.390	0.426

*Cluster robust standard error in parenthesis*.

### Endogeneity Test

Although in the previous analysis we include a series of control variables that affect healthcare environment and control for fixed effects, which mitigate the endogeneity problem. It is also essential to consider the endogenous problems between the high-speed rail opening and healthcare environment. To avoid possible endogeneity and serial correlation in the errors, this paper adopts historical train stations from 20 years ago as the instrumental variable for 2SLS regression (32). In addition, we add local rail passenger traffic as a control variable for further regression. From the results (Table 5), it is possible to affirm that the model is valid and sturdy.

**Table 5:** Endogeneity test.

**Variable**	**Use historical train stations as IV**			**Add local rail passenger traffic**		
	**TEC**	**MI**	**MW**	**TEC**	**MI**	**MW**
HSR*TIME	1.081^***^	1.207^***^	0.998^***^	1.114^***^	1.066^***^	0.749^***^
	(0.020)	(0.044)	(0.012)	(0.065)	(0.087)	(0.046)
Control variables	YES	YES	YES	YES	YES	YES
Fixed effects	YES	YES	YES	YES	YES	YES
Obs	2,890	2,890	2,890	2,890	2,890	2,890
R^2^	0.298	0.430	0.366	0.331	0.257	0.270

*Cluster robust standard error in parenthesis. Significance*:

*** *p < 0.01*.

## Conclusions

This paper explores the impact of high-speed railway opening on health care environment applying Difference-in-Differences method (DID) in China. The empirical results highlight that high-speed railway opening has a significant positive effect on public health care environment. And the performance of the eastern region is better than that of the central and western regions. Finally, the article examines the mechanisms by which the opening of high-speed rail affects the health care environment. High-speed rail enhances the health care environment by improving road accessibility and economic development.

The results of the article have important policy implications. Firstly, the opening of the high-speed railway should be used as an opportunity to better 'bring in' and 'go out' medical resources. Specifically, we should strengthen exchanges with developed medical regions to learn about advanced medical technology and management. At the same time, we should also pay attention to the introduction and training of high-level medical talents and resources. Secondly, as there are differences in the level of medical development in different regions, local governments should actively encourage medical cooperation between medical institutions to promote the spatial spillover of the health effects of the opening of the high-speed railway. Thirdly, in addition to directly improving health care conditions, the government should also work to improve the overall living environment in other areas such as economic development and financial development, thereby increasing the locational advantage and attracting the construction of the high-speed rail, which in turn will lead to improved medical conditions. Ultimately, this will lead to a virtuous cycle of improving the health care environment.

## Data Availability Statement

The raw data supporting the conclusions of this article will be made available by the authors, without undue reservation.

## Author Contributions

C-XS: writing the original draft, econometric analyses, and data access. C-XQ: checking the original draft. JL: data access. All authors contributed to the article and approved the submitted version.

## Conflict of Interest

The authors declare that the research was conducted in the absence of any commercial or financial relationships that could be construed as a potential conflict of interest.
